# The Novelty of Orthopedic Rehabilitation After Conservative Management for Patellar Dislocation With Partial Tear of Medial Meniscus and Early Osteoarthritis in a 31-Year-Old Female

**DOI:** 10.7759/cureus.46298

**Published:** 2023-09-30

**Authors:** Pooja R Tiwari, Deepali S Patil, Anam R Sasun, Pratik Phansopkar

**Affiliations:** 1 Department of Musculoskeletal Physiotherapy, Ravi Nair Physiotherapy College, Datta Meghe Institute of Higher Education & Research, Wardha, IND

**Keywords:** physiotherapy, conservative management, medial patellofemoral ligament, patellofemoral joint, patellar dislocation

## Abstract

Primary patellar dislocation or first-time patellar dislocation is the second most frequent cause of knee injuries which overall accounts for about 3% of other knee injuries. The patellofemoral joint is formed by the patella connecting to the femoral trochlea and creates both static and dynamic structures of the knee. There are basically three types of patellar dislocation: superior, lateral, and medial. The lateral dislocation is the most frequent one. Females are more vulnerable and are at higher risk than males. Muscular weakness or muscular imbalance leads to patellar instability, and ultimately to dislocation. The recurrence rate after primary patellar dislocation is 15-60%. This case report is of a 31-year-old female with patella dislocation with a medial meniscal tear and a case of early osteoarthritis for whom we planned goal-oriented physiotherapy rehabilitation week-wise and progressed every week. The assessment was taken before and after physiotherapy rehabilitation. The patient was managed conservatively with a long knee brace, and physiotherapy started after one month. Due to prolonged immobilization, the patient suffered from quadriceps muscle atrophy. The physiotherapist focused on biomechanism and got the expected results in pain reduction, regaining strength, and improving range of motion, and the patient was able to walk properly without taking any support after rehabilitation.

## Introduction

The second most frequent cause of traumatic hemarthrosis of the knee is acute traumatic patellar dislocation, following anterior cruciate ligament tear, and accounts for 3% of all traumatic knee lesions [1,2]. The patellofemoral joint is comprised of both static and dynamic structures for stabilization around the knee joint. The two patellar joint surfaces lateral and medial are congruent and symmetric with the femoral trochlea [3]. The morphology remains the same but after some age cartilage becomes thinner and creates a hollowness in the middle. The medial patellafemoral ligament (MPFL) and vastus medialis oblique (VMO) play a crucial for the stability of the joint [4].

The dislocation is common in any sports injury or by external tibia rotation with a fixed foot on the ground. Individuals with ligamentous laxity are also prone to this type of dislocation [5]. Females are more affected and are at higher risk than males. Muscular weakness or any imbalance between muscles can also lead to patellar instability further leads to dislocation. The recurrence rate after primary patellar dislocation is 15-60% [6].

There are three types of patellar dislocation: lateral dislocation, medial dislocation, and superior dislocation. From which lateral dislocation is more common, medial and superior are least common. Superior dislocation is rare but tends to occur in the aging population [7] and is always confused with patellar tendon rupture as it occurs after forceful quadriceps contraction, with or without knee hyperextension. Patellar dislocations are defined as either intra-articular (rotation on the axial or vertical axis) or extra-articular (tendon rupture in addition to rotation) [8,9].

There are various options for the management of patellar dislocation starting with conservative management followed by physiotherapy and for more serious dislocation surgical management followed by physiotherapy. The physiotherapist evaluates and plans the therapies for patients. They work on pain, swelling, and subsiding inflammation, and then focus on range of motion and joint flexibility. The next step is to improve muscular strength and the last focus is on coordination or training proprioception exercises [10].

## Case presentation

**Figure 1:**
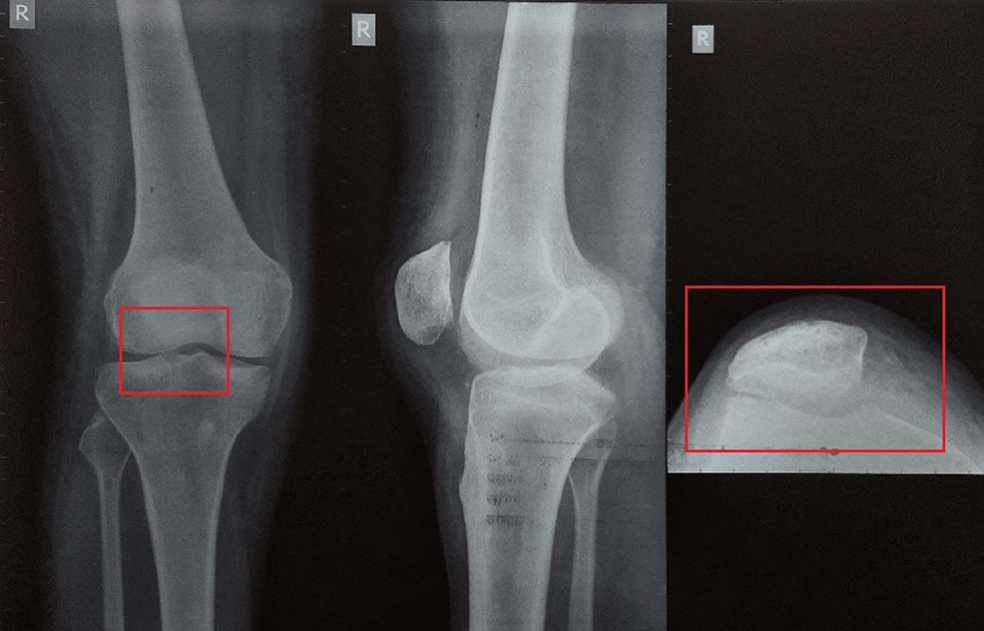
X-ray of the anterior aspect of knee shows lateral dislocation of patella.

**Figure 2:**
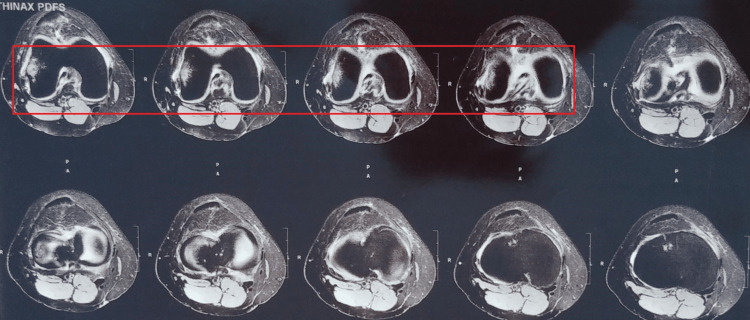
MRI of partial tears of popliteus tendon and anterior horn of the lateral meniscus.

**Figure 3:**
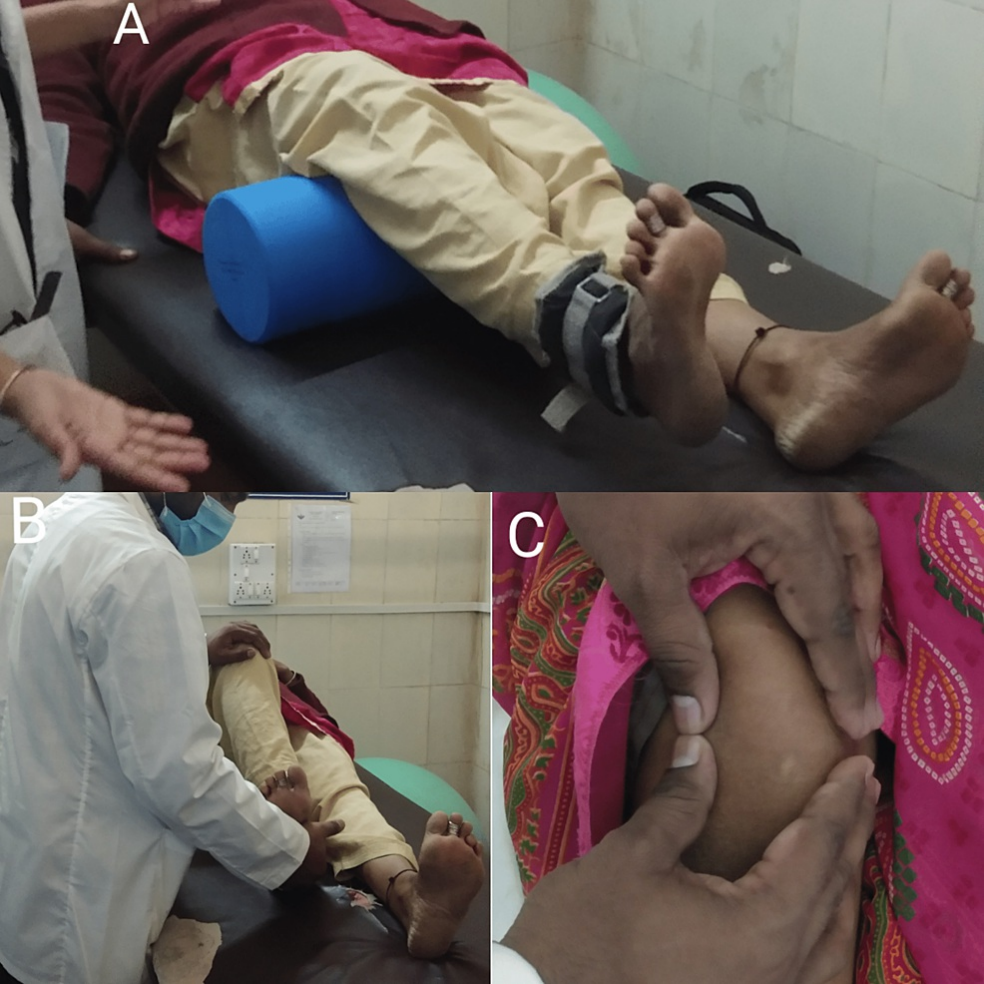
A. VMO strengthening, B. The therapist applies manual resistance in VMO strengthening and C. Patellar mobilization VMO: vastus medialis oblique

A 31-year-old female presented to the orthopedic OPD with major complaints of pain, edema, a shift of the patella to the lateral direction, and an inability to correctly straighten her right leg since one day. The pain was sudden at the onset and gradually progressive in nature. The pain aggravated on movements or position changes and relieved on rest and medication. The patient gave a history of falling from stairs one day back after which she was managing pain and swelling at home. After all physical examinations, the examiner suggested the patient for an X-ray of the anterior aspect of the knee joint and magnetic resonance imaging (MRI) of the knee joint. The X-ray and MRI findings are shown in Figures 1, 2. The patient was managed by giving a long knee brace and advised physiotherapy but the patient kept the limb immobilized for one month and came for physical therapy shown in Figure 3 after a month. The therapeutic interventions are given in Tables 1, 2.

**Table 1:** Week-wise therapeutic interventions

Day/ Week	Precautions	Range of motion	Strength	Functional Activities
Day 1 to week 1	Avoid passive range of motion.	Active knee range of motion can be achieved in a sitting position.	No strengthening	Full weight bearing during transfers and ambulation using assistive devices.
2 to 3 weeks weeks	Avoid passive range of motion	Active knee flexion with no weight bearing	None	Full weight bearing during ambulation and transfers
4 to 6 weeks	Maintain knee immobilizer if tenderness is present.	Active range of motion knee for flexion/extension	Isometrics exercises to quadriceps and hamstrings.	Full weight bearing during ambulation and transfers. Remove immobilizer for level ground walking
8 to 12 weeks	None	Both active and passive ranges of motion	Progressive resistive exercises to quadriceps and hamstrings with weight or manual resistance, plyometric closed chain exercises.	Full weight bearing during ambulation and transfer without assistive devices.

**Table 2:** Goal-oriented physiotherapy rehabilitation VMO: vastus medialis oblique, ROM: range of motion

Sr.no	Goals	Rehabilitation	Regimen
1.	To reduce pain, effusion, and inflammation.	Ultrasound modality using 1 MHz frequency and cryotherapy	Once a day for 10 minutes for the ultrasound. Thrice a day for 8-10 mins for cryotherapy.
2.	To regain possible flexion-extension ROM of the knee.	The affected limb is kept immobilized for 4 weeks after conservative management. So, we avoid flexion and extension passive ROMs. Assisted Straight leg raises (SLR) and active ankle-toe movements. After 4 weeks knee ROM can be initiated	Starting with 10 reps × 1 set can be progressed to 10 reps × 2 sets once the pain gets subside.
3.	To regain the strength of lower leg muscles	As soon as possible the gentle isometrics of the lower limb should be started focusing more on the quadriceps and hamstrings muscles. Also, on VMO muscle.	10 reps × 1 set with 5 secs hold
4	To prevent future injuries	Proprioception training exercises such as tandem walking, sideways walking, step-up and step-down, and balancing exercises.	10 reps × 1 set
5.	To improve mobility	Manual Patellar Mobilizations. For pain Maitland grade 1 and 2 For increase in ROM Maitland grade 3 and 4	Once a day was given by the therapist
6.	To make the patient functionally independent	Ambulation using full weight bearing or gait training	Thrice a day (1 round)

Clinical findings

The patient was conscious, orientated, cooperative, and hemodynamically stable. On observation the patient is mesomorphic, seen in a supine lying position with both the lower legs in an extended position, muscle wasting of the quadriceps is seen, and the swelling is present over the superior and anterior aspects of the right knee. On palpation, Grade II tenderness with a slight temperature rise, and boggy swelling are present over the anterior aspect of the right knee, the motor assessment and outcomes are given in the outcome measure (Tables 3-5). The special tests like the McMurray test, Apley’s test, and Clarke’s test were positive.

**Table 3:** For pain and functional stability

Outcome measures	First day of physiotherapy	After eight weeks of physiotherapy	Follow-up after one week
Numeric pain rating scale (NPRS)	7/10	2/10	1/10
Lysholm score	64	74	84
Kujala score (Anterior knee pain scale)	15%	69%	82%

**Table 4:** Range of motion of the affected right leg

Range of motion	Before physiotherapy	After eight weeks of physiotherapy	Follow-up after one week
Hip Flexion	0°-20°	0°-60°	0°-80°
Extension	0°-10°	0°-20°	0°-20°
Abduction	0°-30°	0°-40°	0°-45°
Knee Flexion	0°-20°	0°-50°	0°-70°
Extension	20°-5°	50°-5°	70°- 0°
Ankle Dorsiflexion	0°-15°	0°-20°	0°-20°
Planterflexion	0°-40°	0°-45°	0°-50°

**Table 5:** Manual muscle testing of the affected right leg

Manual Muscle Testing	First day of physiotherapy	After eight weeks of physiotherapy	Follow-up after one week
Hip flexors	2/5	3/5	4/5
Extensors	2/5	3/5	4/5
Abductors	2/5	3/5	4/5
Knee flexors	2/5	3/5	4/5
Extensors	2/5	3/5	4/5
Ankle dorsiflexion	3/5	4/5	4/5
Ankle platerflexion	3/5	4/5	4/5

## Discussion

Lateral patellar dislocation is a common dislocation seen among individuals. This type of dislocation is common because of the slight pull of the quadriceps muscle from the mechanical axis of a limb. The quadriceps angle (Q-angle) is a line from the anterior superior iliac spine (ASIS) to the mid patella and another line from the tibial tubercle to the mid patella. The normal range is 15°. The increased Q-angle is more likely to develop patellar dislocation. The primary static stabilizer is the MPFL which gets injured in dislocation and makes it difficult to flex the knee up to 20° [5]. A study conducted in 2020 was a meta-analysis and randomized control trial that concluded that despite high recurrent rates of patellar dislocation conservative management followed by physiotherapy showed better results and produced better outcomes than surgical management [11]. According to the review done in 2010, a variety of different physiotherapy strategies for the rehabilitation of patients following lateral patellar dislocation have been reported. The best strategy is still unknown. As a result, to improve the management of this complex musculoskeletal condition, a well-designed randomized controlled trial is required to determine how best to manage patients following a lateral patellar dislocation [12]. To manage a patient group, generic lower limb assessment and treatment strategies are widely used. Given the scarcity of previous research in this area, more research is now needed to assess the efficacy of these interventions and provide physiotherapists with evidence to support their management strategies [13]. Nonoperative and postoperative rehabilitation should focus on resolving pain and edema, restoring motion, and incorporating isolated and multijoint progressive strengthening exercises targeting the hip and knee. Before returning to sports, both functional and isolated knee strength measurements should be used to determine leg symmetry strength, as well as patient-reported outcome measures to assess the patient's perceived physical abilities and patellofemoral joint stability [14]. A study was conducted in 2011 to compare both operative and non-operative surgical management for primary patellar dislocation. The study selected 39 patients who were arranged randomly into two groups. The first group was managed by immobilization and physiotherapy and the second group by MPFL reconstruction followed by physiotherapy. The outcome was measured by using the Kujala score, the second group showed a better result with a follow-up of two years [15]. A study conducted in 2019 to know early functional rehabilitation after the dislocation of the patella found that the conservative treatment protocols were more restrictive in terms of weight bearing, ROM, and orthosis use in the first weeks following trauma, whereas after surgery more functional settings were used. Overall, functional treatment strategies were initiated earlier after MPFL reconstruction than after conservative treatment which reduces the risk of limitation and future recurrent dislocations [16].

## Conclusions

Conservative management followed by physiotherapy rehabilitation was effective in primary patellar dislocation. The physiotherapist was able to reduce pain, improve strength and range of motion, and achieve better functional stability. As there is about a 15-60% chance of getting recurrent dislocation of the patella after primary dislocation, medial patellofemoral surgery will be a good option followed by physiotherapy and functional strategies can be initiated to avoid further future risk.

## References

[1] Harilainen A, Myllynen P, Antila H, Seitsalo S (1988). The significance of arthroscopy and examination under anaesthesia in the diagnosis of fresh injury haemarthrosis of the knee joint. Injury.

[2] Stefancin JJ, Parker RD (2007). First-time traumatic patellar dislocation: a systematic review. Clin Orthop Relat Res.

[3] Duthon VB (2015). Acute traumatic patellar dislocation. Orthop Traumatol Surg Res.

[4] Hinton RY, Sharma KM (2003). Acute and recurrent patellar instability in the young athlete. Orthop Clin North Am.

[5] Hayat Z, El Bitar Y, Case JL (2022). Patella dislocation. StatPearls.

[6] Fithian DC, Paxton EW, Stone ML, Silva P, Davis DK, Elias DA, White LM (2004). Epidemiology and natural history of acute patellar dislocation. Am J Sports Med.

[7] Yip DK, Wong JW, Sun LK, Wong NM, Chan CW, Lau PY (2004). The management of superior dislocation of the patella with interlocking osteophytes--an update on a rare problem. J Orthop Surg (Hong Kong).

[8] Ofluoglu O, Yasmin D, Donthineni R, Yildiz M (2006). Superior dislocation of the patella with early onset patellofemoral arthritis: a case report and literature review. Knee Surg Sports Traumatol Arthrosc.

[9] Respizzi S, Cavallin R (2014). First patellar dislocation: from conservative treatment to return to sport. Joints.

[10] Clift RK, El-Alami W (2012). Superior patellar dislocation: the value of clinical examination and radiological investigation. BMJ Case Rep.

[11] Xing X, Shi H, Feng S (2020). Does surgical treatment produce better outcomes than conservative treatment for acute primary patellar dislocations? A meta-analysis of 10 randomized controlled trials. J Orthop Surg Res.

[12] Smith TO, Davies L, Chester R, Clark A, Donell ST (2010). Clinical outcomes of rehabilitation for patients following lateral patellar dislocation: a systematic review. Physiotherapy.

[13] Smith TO, Chester R, Clark A, Donell ST, Stephenson R (2011). A national survey of the physiotherapy management of patients following first-time patellar dislocation. Physiotherapy.

[14] Watson R, Sullivan B, Stone AV, Jacobs C, Malone T, Heebner N, Noehren B (2022). Lateral patellar dislocation: a critical review and update of evidence-based rehabilitation practice guidelines and expected outcomes. JBJS Rev.

[15] Bitar AC, Demange MK, D'Elia CO, Camanho GL (2012). Traumatic patellar dislocation: nonoperative treatment compared with MPFL reconstruction using patellar tendon. Am J Sports Med.

[16] Hilber F, Pfeifer C, Memmel C (2019). Early functional rehabilitation after patellar dislocation-what procedures are daily routine in orthopedic surgery?. Injury.

